# Six-month real-world glycemic improvement after Smart MDI system in type 1 diabetes

**DOI:** 10.1210/jendso/bvag244

**Published:** 2026-10-28

**Authors:** Giada Rossi, Ida Pastore, Laura Montefusco, Lorenzo Pozzi Canova, Francesca D’Addio, Paolo Fiorina

**Affiliations:** International Center for T1D, Pediatric Clinical Research Center Romeo ed Enrica Invernizzi, DIBIC, Università degli Studi di Milano, 20157 Milan, Italy; International Center for T1D, Pediatric Clinical Research Center Romeo ed Enrica Invernizzi, DIBIC, Università degli Studi di Milano, 20157 Milan, Italy; Division of Endocrinology, ASST Fatebenefratelli Sacco, 20157 Milan, Italy; Division of Endocrinology, ASST Fatebenefratelli Sacco, 20157 Milan, Italy; International Center for T1D, Pediatric Clinical Research Center Romeo ed Enrica Invernizzi, DIBIC, Università degli Studi di Milano, 20157 Milan, Italy; International Center for T1D, Pediatric Clinical Research Center Romeo ed Enrica Invernizzi, DIBIC, Università degli Studi di Milano, 20157 Milan, Italy; Division of Endocrinology, ASST Fatebenefratelli Sacco, 20157 Milan, Italy; International Center for T1D, Pediatric Clinical Research Center Romeo ed Enrica Invernizzi, DIBIC, Università degli Studi di Milano, 20157 Milan, Italy; Division of Endocrinology, ASST Fatebenefratelli Sacco, 20157 Milan, Italy; Nephrology Division, Boston Children's Hospital, Harvard Medical School, Boston, 02115 MA, USA; Transplantation Research Center, Brigham and Women's Hospital, Harvard Medical School, Boston, 02115 MA, USA

**Keywords:** type 1 diabetes, multiple daily injections, smart insulin pen, continuous glucose monitoring, carbohydrate counting

## Abstract

**Background and Aims:**

Smart multiple daily injection (Smart MDI) system integrating continuous glucose monitoring (CGM), insulin pens, and decision-support tools may optimize glycemic control in type 1 diabetes (T1D). However, real-world data remain limited. We evaluated 6-month glycemic outcomes following transition to Smart MDI in adults with T1D and explored differences according to carbohydrate counting status.

**Methods:**

This retrospective, single-center, observational study included 24 adults with T1D on MDI who transitioned to Smart MDI. CGM metrics were analyzed at baseline and after 1, 3, and 6 months. The primary outcome was change in time in range (TIR 70-180 mg/dL). Secondary outcomes included time above range (TAR), time below range (TBR), mean glucose, glucose management indicator (GMI), coefficient of variation, and HbA1c. Exploratory analyses compared individuals practicing carbohydrate counting (n = 12) with those who did not (n = 12).

**Results:**

TIR increased progressively from 56.1 ± 20.4% to 66.3 ± 17.5% (median change +9.0%, *P* < .01). TAR and TBR significantly decreased. HbA1c declined from 7.6 ± 1.1% to 7.3 ± 0.9% (median change −0.2%, *P* = .01). While between-group differences were not significant, individuals not practicing carbohydrate counting showed preliminary within-group improvements in TIR, TAR, mean glucose, GMI, and HbA1c, without increased TBR, whereas changes in the carbohydrate counting group were smaller.

**Conclusions:**

Transition to the Smart MDI system was associated with clinically meaningful improvements in CGM metrics and HbA1c over 6 months. Improvements among individuals not practicing carbohydrate counting suggest that the Smart MDI system may provide standardized decision support also beneficial in less structured self-management settings. Larger studies are warranted to confirm these findings.

Multiple daily injections (MDI) insulin therapy continues to be the most common treatment for type 1 diabetes mellitus (T1D) worldwide, particularly in Europe (95.4% in Eastern Europe, 56.5% in Western Europe, compared with 37% in the United States) (1, 2). Continuous glucose monitoring (CGM) has improved the time spent in the glucose range in patients with T1D on MDI therapy. Particularly, an increase from 45% to 60% of time in range (TIR) was observed in T1D patients using real-time CGM compared with those using self-monitoring of blood glucose (3, 4). However, the internationally established target of TIR >70% is hardly reached with CGM stand-alone due to the limitations of the system itself (5, 6). Indeed, missed insulin doses, hypoglycemia episodes due to insulin accumulation, or mismatched bolus and carbohydrate intake are common issues observed in maintaining glycemic control with the sole use of CGM (7, 8). Sensor-augmented pumps and automated insulin delivery systems are highly effective in overcoming these concerns, but limitations in their widespread use are still present to date, mostly due to limited access, high costs, patients’ perceived complexity, and acceptance (9-12). Smart insulin pens, integrated with CGM and decision-support apps, provide a practical tool for optimizing insulin dosing in MDI-treated patients who do not have access to other systems (13, 14). Recent real-world evidence, including data from over 1800 adults with T1D using the Smart MDI system, combining the CGM Simplera™ sensor and the smart insulin pen, InPen™, suggests that timely engagement with system alerts may improve glycemic control (15). However, high-quality clinical data are still lacking, and more evidence is needed to compare the effects of Smart MDI and other insulin delivery and glucose monitoring systems on glycemic and patient-reported outcomes (16). This study aims to investigate the effects of the Smart MDI system on glucose control over time in a cohort of adult patients with T1D on MDI therapy. In addition, given the established role of carbohydrate counting in optimizing prandial insulin dosing and improving glycemic outcomes in T1D (17), we compared glycemic outcomes between patients performing carbohydrate counting and those not applying this strategy.

## Materials and methods

### Study design

We conducted a real-world, observational, retrospective, non-interventional, single-center study in adults with T1D who initiated the Smart MDI system as part of routine clinical care at our tertiary diabetes outpatient clinic of “Luigi Sacco” Hospital between January 2024 and March 2025. The decision to initiate Smart MDI therapy was made by the treating clinical team based on routine clinical practice. All participants had received appropriate training in the use of CGM and underwent regular clinical follow-up. Eligible participants were adults aged ≥18 years with a diagnosis of T1D for at least 1 year, HbA1c <10% (86 mmol/mol) at baseline, previous use of a certified and approved CGM, and treatment with multiple daily injections before initiation of Smart MDI. Exclusion criteria were non-autoimmune diabetes, pregnancy, estimated glomerular filtration rate <60 mL/min/1.73 m^2^, episodes of severe hypoglycemia or diabetic ketoacidosis within the previous 6 months, hospital admission for acute illness within the previous 3 months, corticosteroid therapy within the previous 3 months, or known allergy to any component of the CGM system. Clinical and laboratory data were retrospectively obtained from patients’ medical records, while CGM-derived metrics were obtained from CGM reports of the specific sensor. Baseline data (T0) included demographic and diabetes-related clinical characteristics, together with CGM metrics derived from the predefined 14-day period preceding Smart MDI initiation. Follow-up CGM outcomes were assessed using predefined 14-day windows at 1 (T1), 3 (T2), and 6 (T3) months after Smart MDI initiation. These time points were defined for the purpose of the retrospective data analysis and did not necessarily correspond to study-specific clinical visits. Clinical follow-up and HbA1c measurement were performed as part of routine care at a 6-month visit. Additional interim assessments by the diabetologist, the dietitian, and/or diabetes specialist nurse were performed according to individual clinical needs. The primary outcome was to assess significant differences in glycemic outcomes with the Smart MDI system compared with the previous MDI therapy without sensor integration. This was measured by the percentage of TIR within the CGM range of 70 to 180 mg/dL. Secondary endpoints included overall improvements in mean glucose levels, percentage of time below 70 mg/dL (TBR1), percentage of time below 54 mg/dL (TBR2), percentage of time above 180 mg/dL (TAR1), percentage of time above 250 mg/dL (TAR2), mean sensor glucose (SG), glucose management indicator (GMI), and coefficient of variation based on CGM metrics, and reduction in HbA1c values after 6 months of use of the Smart MDI system. A prespecified subgroup analysis was performed according to carbohydrate (CHO) counting practices before Smart MDI initiation. Carbohydrate-counting status was determined clinically by a dietitian specialized in T1D management, based on the participant's usual approach to carbohydrate counting for mealtime insulin dosing. Patients who regularly counted carbohydrates as part of their previous insulin dosing strategy were classified as the CHO-counting group, whereas those who did not routinely count carbohydrates were classified as the non-CHO-counting group. Changes in CGM metrics and HbA1c were evaluated within and between the two groups over the study period. All participants provided written informed consent for the collection and use of clinical data for research purposes, according to applicable institutional requirements.

### Smart MDI system and clinical management

The Smart MDI system consisted of the Bluetooth-connected InPen™ smart insulin pen, Simplera™ CGM system, and the InPen™ mobile application. The system integrates CGM-derived glucose data with insulin dosing information and provides insulin dose-calculation support and actionable alerts. Insulin doses administered through the InPen™ are automatically recorded in the application, while CGM data are integrated into the platform. The system also provides alerts to support timely mealtime insulin administration and correction of missed boluses, and elevated or low glucose levels. All the participants in the present study transitioned to the Simplera™ CGM system concomitantly with initiation of InPen™. At initiation, participants received training from a diabetes specialist nurse, including CGM sensor placement, installation and activation of the mobile application and the smart insulin pen, and practical instruction on the use of the Smart MDI system. A telephone follow-up was performed approximately 1 week after initiation, and further technical or clinical support was provided according to individual needs by the diabetes specialist nurse, diabetes specialist dietitian, and the treating diabetologist. During follow-up, insulin therapy was managed according to routine clinical practice. Minor insulin adjustments could be made by participants based on recommendations provided by the Smart MDI system, whereas additional adjustments were made by the dietitian and/or treating physician when clinically indicated.

### Statistical analysis

Statistical analyses were performed with support from the Medtronic Statistical Service (StatS), according to a prespecified Statistical Analysis Plan (SAP). The investigators provided the study concept and anonymized/de-identified clinical dataset, while Medtronic Statistical Service developed the SAP and performed the statistical analyses. The authors were responsible for the clinical interpretation of the results. Continuous variables are reported as mean ± standard deviation for descriptive purposes. Changes in glycemic outcomes over time are presented as median with interquartile range (IQR). Categorical variables are expressed as counts and percentages. For overall and within-group comparisons of glycemic outcomes across periods, the non-parametric Wilcoxon signed-rank test was employed. Comparisons between the non-carbohydrate counting and carbohydrate counting groups were conducted using the non-parametric Wilcoxon rank-sum test. Median within-patient differences, IQRs, and 95% confidence intervals for the Hodges-Lehmann estimate (HL 95% CI) of the location shift were reported for each paired comparison. Missing values were not imputed; analyses were performed using the available data at each time point, with only participants with non-missing observations at both time points included in each paired comparison. For the primary endpoint (TIR), effect size was quantified using Cohen's *d* for paired observations. To address sample size constraints, post hoc statistical power and the minimum detectable effect size (MDES) for a target power of 80% (α = .05, 2-tailed) were calculated for the longitudinal primary outcome analysis. All tests were 2-sided, and *P*-values <.05 were considered statistically significant. Statistical analyses were conducted using R statistical software version 4.5.2 and Prism GraphPad software version 10.4.0 (527).

## Results

### Patients’ characteristics

A total of 30 adults with T1D were assessed for eligibility during the study period. Six individuals were excluded before study inclusion: one did not meet the predefined eligibility criteria because of HbA1c >10%, while four declined to initiate the Smart MDI system, and one was a first-time glucose sensor user. Therefore, 24 participants initiated the Smart MDI system. Follow-up CGM data were available for 24 participants at 1 month, 22 at 3 months, and 20 at 6 months and were included in the analysis. The reasons for unavailable follow-up data are detailed in Fig. S1 (18), which illustrates the overall cohort selection and availability of follow-up data. All participants had been using a CGM sensor prior to switching to the Smart MDI system: 19/24 wore the FreeStyle Libre 2 sensor, 3/24 the Guardian 4 sensor, 1/24 the Dexcom G6 sensor, and 1/24 the Dexcom ONE sensor. Patients were predominantly male (67%), with a mean age of 39.9 ± 15.5 years, a mean diabetes duration of 13.8 ± 10.3 years, and mean HbA1c values of 7.6 ± 1.1% (59.5 ± 12.7 mmol/mol) (Table 1). At study entry, mean time in range (70-180 mg/dL) was 56.1 ± 20.4%, mean time above range (>180 mg/dL) was 40.4 ± 22.7%, and mean time below range (<70 mg/dL) was 3.5 ± 3.8%. CGM data were available for all participants at baseline and 1 month after, for 91.7% at 3 months, and for 83.3% at 6 months. The prevalence of diabetes-related complications and hypoglycemia unawareness was low, whereas nearly one-third of participants had at least one additional autoimmune disease. Half of the study population used to count carbohydrates to calculate meal-time bolus doses (CHO-counting group, n = 12), while the remaining half used fixed insulin doses at meals (non-CHO-counting group, n = 12). Baseline demographic and clinical characteristics were comparable between groups and are summarized in Table 1.

**Table 1:** Baseline demographic and clinical characteristics of the study population

	Total	CHO non-counters	CHO counters	*P* value
N	24	12	12	
M/F—n (%)	16/8 (67/33)	6/6 (50/50)	10/2 (83/17)	.193
Age (years)	39.9 ± 15.5	46.2 ± 15.9	33.4 ± 12.5	.064
BMI (Kg/m^2^)	24.1 ± 3.3	23.6 ± 3.3	24.6 ± 3.4	.528
Diabetes duration (years)	13.8 ± 10.3	15.7 ± 12.2	12 ± 8.1	.385
HbA1c (%) [mmol/mol]	7.6 ± 1.1 [59.5 ± 12.7]	8.0 ± 1.3 [64.1 ± 19.6]	7.2 ± 0.9 [54.9 ± 13.7]	.088
Hypo-awareness—n (%)	22 (92)	12 (100)	10 (83)	.478
Any diabetes-related complication—n (%)	6 (25)	4 (33)	2 (17)	.640
ASCVD—n (%)	2 (8.3)	2 (17)	0 (0)	.478
Diabetic retinopathy—n (%)	2 (8.3)	2 (17)	0 (0)	.478
Diabetic kidney disease—n (%)	3 (13)	2 (17)	1 (8.3)	>.999
Diabetic neuropathy—n (%)	3 (13)	1 (8.3)	2 (17)	>.999
Other autoimmune diseases—n (%)	7 (29) TD 5 (21) CD 1 (4) RA 1 (4)	4 (33) TD 2 (17) CD 1 (8) RA 1 (8)	3 (25) TD 3 (25) CD 0 (0) RA 0 (0)	>.999
Other glucose lowering drugs (metformin)	2 (8.3)	1 (8.3)	1 (8.3)	>.999
Previous CGM	24 (100)	12 (100)	12 (100)	>.999
FreeStyle Libre 2	19 (79)	10 (83)	9 (75)	
Guardian 4	3 (13)	1 (8.3)	2 (17)	
Dexcom G6	1 (4.2)	0 (0)	1 (8.3)	
Dexcom ONE	1 (4.2)	0 (0)	1 (8.3)	

Abbreviations: ASCVD, atherosclerotic cardiovascular disease; BMI, body mass index; CD, celiac disease; CGM, continuous glucose monitoring; CHO, carbohydrates; F, females; HbA1c, glycated hemoglobin; M, males; N, number of patients; ns, not significant; RA, rheumatoid arthritis; TD, thyroid disease.

Data are expressed as mean ± standard deviation (SD) and percentages (%) unless otherwise reported. *P* values are considered statistically significant when <.05.

### Glycometabolic control

Overall, TIR increased significantly and progressively during follow-up. Mean TIR rose from 56.1 ± 20.4% at baseline to 62.7 ± 17.4% at 1 month, 65.1 ± 17.0% at 3 months, and 66.3 ± 17.5% at 6 months. The median change from baseline was +5.5% (IQR −0.2, 10.2; HL 95% CI 1.5, 10.5) at 1 month (*P* = .009), +8.0% (IQR 0.2, 17.0; HL 95% CI 2.0, 13.5) at 3 months (*P* = .006), and +9.0% (IQR 2.8, 22.8; HL 95% CI 4.0, 17.5) at 6 months (*P* = .004), demonstrating a sustained increase in time spent within the target glucose range across the study period with the new system (Fig. 1A and B). At 6 months (n = 20), the mean increase in TIR from baseline was +10.8 ± 14.1%, corresponding to a medium-to-large effect size (Cohen's *d* = 0.76). For this primary outcome, the post hoc statistical power achieved was 89.8% (n = 20, α = .05), and the MDES for 80% power at n = 20 was *d* = 0.66. To account for the potential impact of sensor transition, a sensitivity analysis restricted to participants using FreeStyle Libre 2 at baseline (n = 19) confirmed significant improvements in TIR at 1, 3, and 6 months, with a median increase of 8.0% at 6 months (IQR, 3.0-22.0; *P* = .011), consistent with the primary analysis. Exposure to hyperglycemia decreased over time. Mean TAR declined from 40.4 ± 22.7% at baseline to 32.1 ± 18.4% at 6 months (Fig. 1C). Time above range >250 mg/dL (TAR2) also decreased, with a median reduction of −2.0% (IQR −6.0, 0.5; HL 95% CI −12.5, −0.5) at 6 months (*P* = .036). Improvements in hyperglycemia exposure were not accompanied by increased hypoglycemia. On the contrary, TBR decreased from 3.5 ± 3.8% at baseline to 1.6 ± 2.1% at 6 months, with median reductions from baseline of −1.0% both at 3 months (IQR −3.8, 0.0; HL 95% CI −5, −0.5; *P* = .020) and at 6 months (IQR −3.0-0.0; HL 95% CI −5.5, −1.0; *P* = .027) (Fig. 1D). Time spent below 54 mg/dL (TBR2) remained low throughout follow-up, with no statistically significant increase compared with baseline. Neither episodes of diabetic ketoacidosis nor episodes of severe hypoglycemia occurred during the study. A comprehensive overview of the changes in the principal CGM metrics with the Smart MDI system over time is shown in Fig. 1A. Considering the entire study population, HbA1c levels significantly decreased from a mean of 7.6 ± 1.1% (59.5 ± 12.7 mmol/mol) at baseline to 7.3 ± 0.9% (56.0 ± 10.1 mmol/mol) at 6 months, corresponding to a median reduction of −0.2% (IQR −0.6, 0.0; HL 95% CI −0.7, −0.1; *P* = .016) (Table 2).

**Figure 1:**
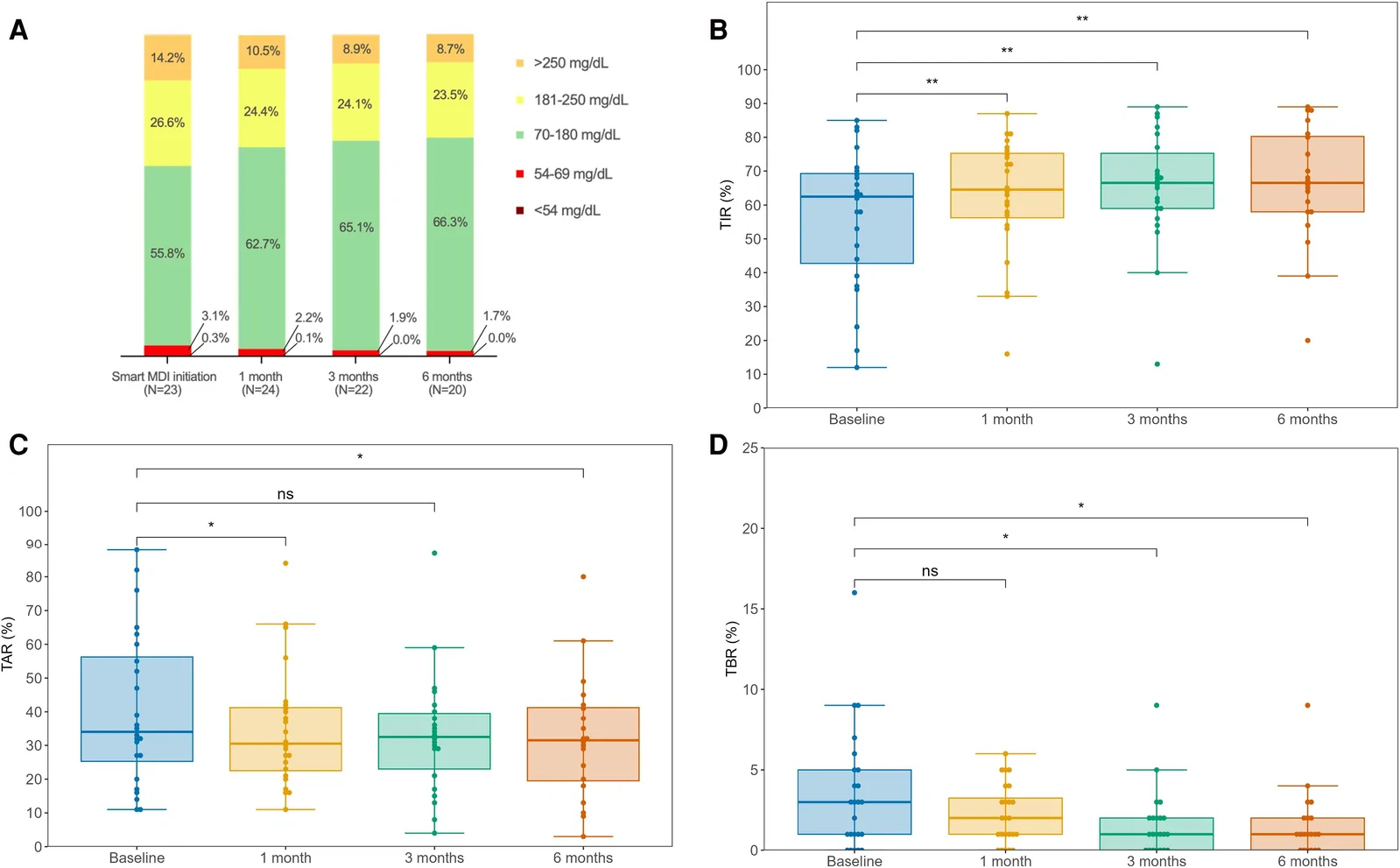
(A-D) Variation in the main CGM metrics (TIR, TAR, and TBR) over time with the use of the Smart MDI system. Note to Fig. 1A. At baseline, TIR and TBR1 percentages slightly differ from the tabulated values in Table 2. In Fig. 1A, percentages were calculated as the mean of individual percentages among participants with complete data for all glycemic categories at the corresponding time point, resulting in n = 23 vs n = 24 in Table 2, because one participant had missing TAR1 and TAR2 data at baseline. Abbreviations: TAR, time above range; TBR, time below range; TIR, time in range.

**Table 2:** Glycemic outcomes before and after the switch to the Smart MDI system

Variable	Time point	Mean ± SD	Median difference from baseline (IQR; HL 95% CI)	*P* value
TIR % (70-180 mg/dL)	Baseline (T0)	56.1 ± 20.4		
	1 month (T1)	62.7 ± 17.4	5.5 (−0.2, 10.2; 1.5, 10.5)	.**009**
	3 months (T2)	65.1 ± 17.0	8.0 (0.2, 17; 2, 13.5)	.**006**
	6 months (T3)	66.3 ± 17.5	9.0 (2.8, 22.8; 4, 17.5)	.**004**
TBR tot % (<70 mg/dL)	Baseline (T0)	3.5 ± 3.8		
	1 month (T1)	2.2 ± 1.9	0 (−2.5, 0; −4.5, 0.5)	.083
	3 months (T2)	1.6 ± 2.1	−1.0 (−3.8, 0; −5, −0.5)	.**020**
	6 months (T3)	1.6 ± 2.1	−1 (−3, 0; −5.5, −1)	.**027**
TBR 1% (54-69 mg/dL)	Baseline (T0)	3.2 ± 3.2		
	1 month (T1)	2.2 ± 1.7	0 (−2, 0; −4, 0.5)	.150
	3 months (T2)	1.9 ± 2.0	−1 (−2.8, 0; −4, 0)	.**031**
	6 months (T3)	1.7 ± 2.1	−0.5 (−3, 0; −5.5, −0.5)	.**037**
TBR 2% (<54 mg/dL)	Baseline (T0)	0.3 ± 0.9		
	1 month (T1)	0.1 ± 0.3	0 (0, 0; −2.5, 0)	.345
	3 months (T2)	0.0 ± 0.0	0 (0, 0; −1, −1)	.089
	6 months (T3)	0.0 ± 0.0	0 (0, 0; −1, −1)	.174
TAR tot % (>180 mg/dL)	Baseline (T0)	40.4 ± 22.7		
	1 month (T1)	35.0 ± 17.9	−4 (−10, 0; −12, 0)	.**049**
	3 months (T2)	33.0 ± 17.9	−4 (−18, 2; −13, 0.5)	.059
	6 months (T3)	32.1 ± 18.4	−6.5 (−22.8, −1; −16.5, −1.5)	.**019**
TAR 1% (181-250 mg/dL)	Baseline (T0)	26.6 ± 10.8		
	1 month (T1)	24.4 ± 8.4	−4 (−5.5, 4.5; −5.5, 2)	.473
	3 months (T2)	24.1 ± 9.1	−1 (−10, 5; −7, 3.5)	.456
	6 months (T3)	23.5 ± 10.5	−3 (−7.5, 4; −8.5, 3)	.286
TAR 2% (>250 mg/dL)	Baseline (T0)	14.2 ± 16.8		
	1 month (T1)	10.5 ± 11.4	0 (−7, 1.5; −9, 1)	.236
	3 months (T2)	8.9 ± 11.0	−2 (−5, 1; −6, 0)	.060
	6 months (T3)	8.7 ± 9.6	−2 (−6, 0.5; −12.5, −0.5)	.**036**
Mean SG (mg/dL)	Baseline (T0)	171.3 ± 39.5		
	1 month (T1)	164.3 ± 28.8	−3.5 (−13.8, 2.5; −17.5, 1)	.102
	3 months (T2)	161.4 ± 27.3	−5.0 (−18.5, 6.5; −17.8, 3.5)	.167
	6 months (T3)	158.9 ± 27.4	−9.5 (−32.5, 5.2; −26.5, 0)	.052
GMI %	Baseline (T0)	7.4 ± 0.9		
	1 month (T1)	7.2 ± 0.7	−0.1 (−0.3, 0.1; −0.4, 0)	.102
	3 months (T2)	7.2 ± 0.7	−0.1 (−0.4, 0.2; −0.4, 0.1)	.167
	6 months (T3)	7.1 ± 0.7	−0.2 (−0.8, 0.1; −0.6, 0)	.052
CV %	Baseline (T0)	34.6 ± 6.0		
	1 month (T1)	33.4 ± 7.6	0.1 (−3.6, 2.4; −2.9, 1.4)	.577
	3 months (T2)	32.6 ± 4.6	−1.4 (−5.2, 1.2; −3.8, 0.7)	.158
	6 months (T3)	32.7 ± 4.8	−1.5 (−4.7, 1.3; −3.9, 1)	.240
HbA1c %; mmol/mol	Baseline (T0)	7.6 ± 1.1; 59.5 ± 12.7		
	1 month (T1)			
	3 months (T2)			
	6 months (T3)	7.3 ± 0.9; 56.0 ± 10.1	−0.2 (−0.6, 0; −0.7, −0.1)	.**016**

Abbreviations: CV, coefficient of variation; GMI, glucose management indicator; HbA1c, glycated hemoglobin; HL 95% CI, 95% confidence intervals for the Hodges-Lehmann estimate; IQR, interquartile range; SD, standard deviation; SG, sensor glucose; TAR, time above range; TBR, time below range; TIR, time in range.

T0 n = 24; T1 n = 24; T2 n = 22; T3 n = 20. Statistically significant *P* values are highlighted in bold.

### Exploratory subgroup analysis by carbohydrate counting status

No statistically significant differences in CGM metrics and HbA1c levels were observed between participants practicing carbohydrate counting and those who did not at any time point (Table 3). Despite the absence of statistically significant between-group differences, within-group analyses revealed a distinct pattern of response over time. Individuals not practicing carbohydrate counting experienced an increase in TIR and a reduction in TAR, mean SG, GMI, and HbA1c levels (Table 3). At 6 months, the median increase in TIR was numerically greater in the non-carbohydrate counting group compared with the carbohydrate counting group [+9.5% (IQR 5.5, 22.0; HL 95% CI 4.5, 22.0) vs +6.5% (IQR 0.5, 21.2; HL 95% CI −4.5, 25.0)], corresponding to a 3% difference in median change (Fig. 2). In contrast, changes observed in the carbohydrate counting group were smaller in magnitude and did not achieve statistical significance within the group, except for reductions in TBR (Table 3).

**Figure 2:**
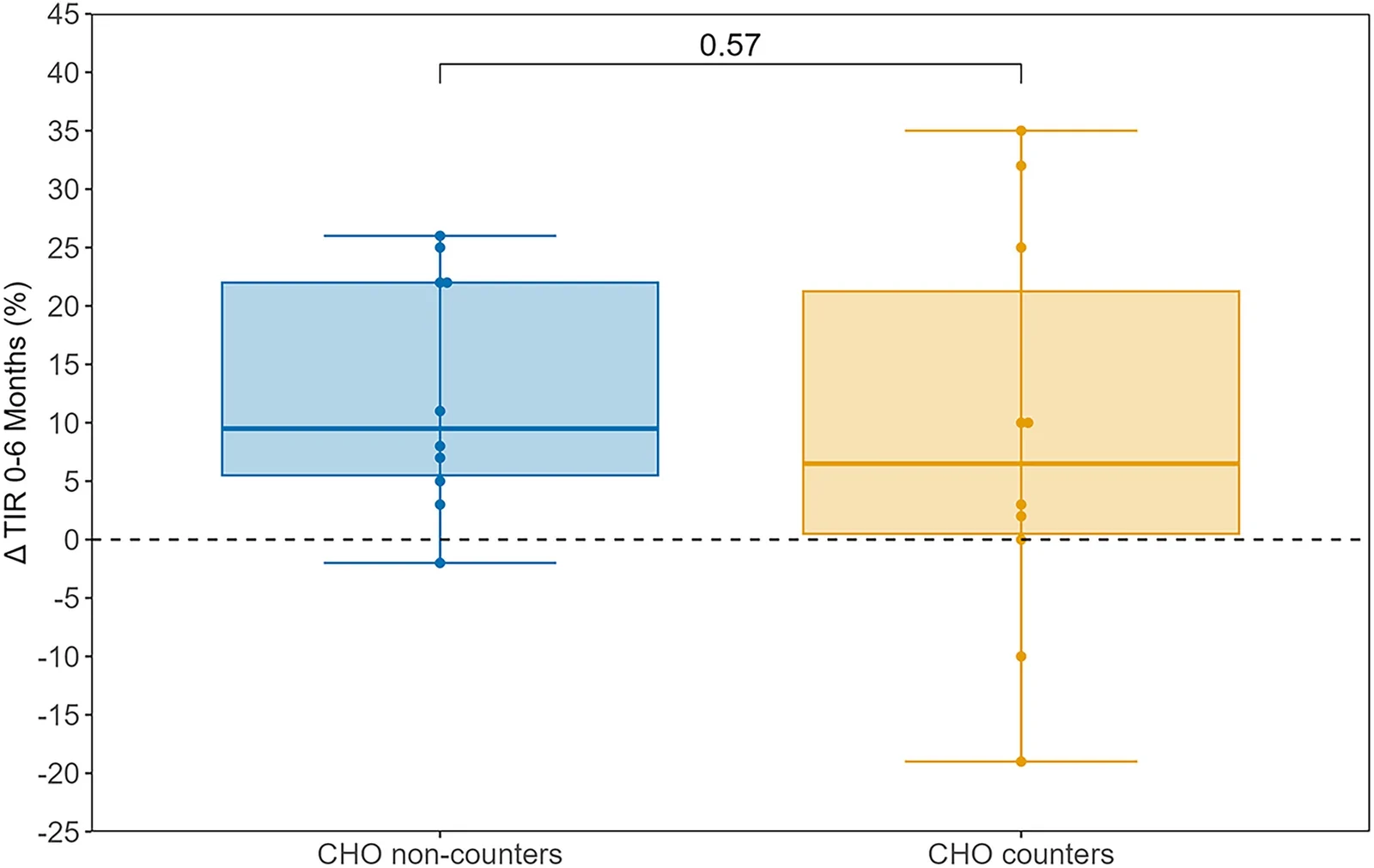
Delta change in time in range after 6 months of Smart MDI system use in CHO non-counters vs CHO counters. Abbreviations: CHO, carbohydrates; TIR, time in range.

**Table 3:** Glycemic outcomes in CHO non-counters vs CHO counters before and after the switch to the Smart MDI system

CHO non-counters (T0 n = 12; T1 n = 12; T2 n = 11; T3 n = 10)					CHO counters (T0 n = 12; T1 n = 12; T2 n = 11; T3 n = 10)					CHO non-counters vs CHO counters
Variable	Time point	mean ± SD	Median difference from baseline (IQR; HL 95% CI)	*P* value difference from baseline	Variable	Time point	mean ± SD	Median difference from baseline (IQR; HL 95% CI)	*P* value difference from baseline	*P* value CHO non-counters vs CHO counters
TIR % (70-180 mg/dL)	Baseline (T0)	50.1 ± 22.0			TIR % (70-180 mg/dL)	Baseline (T0)	62.2 ± 17.4			.166
	1 month (T1)	57.3 ± 19.9	5.5 (1.2, 9.5; 0.5, 14)	.**031**		1 month (T1)	68.2 ± 13.0	5.5 (−1, 11; −2.5, 16)	.119	.157
	3 months (T2)	64.2 ± 22.2	12 (1, 22; 3.5, 22)	.**014**		3 months (T2)	66.0 ± 10.7	4 (−4, 13.5; −4.5, −15)	.185	.693
	6 months (T3)	64.2 ± 22.1	9.5 (5.5, 22; 4.5, 22)	.**008**		6 months (T3)	68.3 ± 12.1	6.5 (0.5, 21.2; −4.5, −25)	.154	.940
TBR tot % (<70 mg/dL)	Baseline (T0)	2.6 ± 2.8			TBR tot % (< 70 mg/dL)	Baseline (T0)	4.4 ± 4.6			.307
	1 month (T1)	1.8 ± 1.5	0 (−2, 0; −4.5, 1)	.343		1 month (T1)	2.7 ± 2.1	−0.5 (−4, 0; −7.5, 1)	.140	.318
	3 months (T2)	1.5 ± 2.6	0 (−3, 0; −7.5, 4.5)	.349		3 months (T2)	1.7 ± 1.5	−1 (−4, −0.5; −6.5, −1)	.**020**	.278
	6 months (T3)	1.9 ± 2.8	−0.5 (−3, 0; −5, 4)	.396		6 months (T3)	1.3 ± 1.2	−1 (−2.8, 0; −8, −1.5)	.**036**	.969
TBR 1% (54-70 mg/dL)	Baseline (T0)	2.5 ± 2.7			TBR 1% (54-70 mg/dL)	Baseline (T0)	3.8 ± 3.6			.334
	1 month (T1)	1.7 ± 1.4	0 (−2, 0; −4.5, 1)	.343		1 month (T1)	2.8 ± 1.9	0 (−1.8, 0; −5.5, 1)	.270	.150
	3 months (T2)	1.8 ± 2.6	0 (−2.5, 0; −6, 4.5)	.348		3 months (T2)	1.9 ± 1.4	−1 (−3, 0; −4.5, 0)	.**042**	.328
	6 months (T3)	2.0 ± 2.7	−0.5 (−2.8, 0; −4.5, 4)	.396		6 months (T3)	1.3 ± 1.2	−0.5 (−2.8, 0; −8, −2)	.059	.875
TBR 2% (<54 mg/dL)	Baseline (T0)	0.1 ± 0.3			TBR 2% (<54 mg/dL)	Baseline (T0)	0.6 ± 1.2			.142
	1 month (T1)	0.1 ± 0.3	0 (0, 0; 0, 0)	>.999		1 month (T1)	0.2 ± 0.4	0 (0, 0; −2.5, −2.5)	.371	.580
	3 months (T2)	0.0 ± 0.0	0 (0, 0; 0,0)	>.999		3 months (T2)	0.0 ± 0.0	0 (−0.5, 0; −1, −1)	.174	>.999
	6 months (T3)	0.0 ± 0.0	0 (0, 0; 0,0)	>.999		6 months (T3)	0.0 ± 0.0	0 (0, 0; −2.5, −2.5)	.371	>.999
TAR tot % (> 180 mg/dL)	Baseline (T0)	47.3 ± 23.8			TAR tot % (> 180 mg/dL)	Baseline (T0)	33.4 ± 20.1			.119
	1 month (T1)	41.0 ± 20.6	−5.5 (−9.2, 0; −19, 0.5)	.102		1 month (T1)	28.9 ± 13.0	−4 (−11.8, 1.2; −16, 5)	.266	.141
	3 months (T2)	33.9 ± 23.6	−9 (−21, 0.5; −21, −1)	.**045**		3 months (T2)	32.1 ± 10.9	−3 (−13.5, 6; −11.5, 8.5)	.656	.768
	6 months (T3)	33.8 ± 23.8	−8 (−21.8, −4.2; −22, −2.5)	.**012**		6 months (T3)	30.4 ± 12.0	−2 (−21.2, 1.2; −21, 11.5)	.358	.850
TAR 1% (180-250 mg/dL)	Baseline (T0)	27.3 ± 6.8			TAR 1% (180-250 mg/dL)	Baseline (T0)	25.9 ± 13.8			.643
	1 month (T1)	26.5 ± 7.5	0 (−4, 5; −6.5, 5.5)	.798		1 month (T1)	22.3 ± 9.0	−4.5 (−6.5, 0.2; −7.5, 2.5)	.254	.271
	3 months (T2)	23.4 ± 10.7	1 (−9.5, 4.5; −10, 5.5)	.683		3 months (T2)	24.8 ± 7.6	−1 (−7.5, 4.5; −10, 9)	.553	>.999
	6 months (T3)	23.0 ± 13.2	−3 (−6, 6; −11, 6)	.859		6 months (T3)	23.9 ± 7.5	−3 (−10.8, 1.2; −14.5, 6.5)	.262	>.999
TAR 2% (>250 mg/dL)	Baseline (T0)	21.5 ± 21.3			TAR 2% (> 250 mg/dL)	Baseline (T0)	7.5 ± 7.4			.090
	1 month (T1)	14.5 ± 14.3	−2 (−12, 2; −15.5, 2)	.152		1 month (T1)	6.6 ± 5.7	0.5 (−1.8, 1; −8, 3)	.892	.131
	3 months (T2)	10.5 ± 15.0	−3 (−5.5, −1.2; −18, −1.5)	.**006**		3 months (T2)	7.3 ± 4.6	1 (−4.5, 2.5; −5, 3)	>.999	.693
	6 months (T3)	10.8 ± 12.5	−5 (−18, 0; −24.5, 0)	.**042**		6 months (T3)	6.5 ± 5.3	−0.5 (−5.2, 1.8; −11, 6)	.514	.879
Mean SG (mg/dL)	Baseline (T0)	185.0 ± 45.0			Mean SG (mg/dL)	Baseline (T0)	157.6 ± 28.8			.112
	1 month (T1)	173.9 ± 34.4	−6.2 (−14.8, 0.2; −27, 3.8)	.126		1 month (T1)	154.6 ± 18.5	0 (−13, 2.8; −23.3, 16)	.636	.126
	3 months (T2)	163.5 ± 36.8	−6 (−27.5, −1.5; −35, −1)	.**032**		3 months (T2)	159.2 ± 14.3	5 (−14, 11; −14, 15.5)	>.999	.948
	6 months (T3)	161.9 ± 35.9	−18.2 ± (−44, 1.5; −40.2, −1)	.**032**		6 months (T3)	155.9 ± 16.8	−5 (−26.8, 5; −27.3, 18)	.507	.821
GMI %	Baseline (T0)	7.7 ± 1.1			GMI %	Baseline (T0)	7.1 ± 0.7			.122
	1 month (T1)	7.5 ± 0.8	−0.1 (−0.4, 0; −0.6, 0.1)	.126		1 month (T1)	7.0 ± 0.4	0 (−0.3, 0.1; −0.5, 0.4)	.636	.126
	3 months (T2)	7.2 ± 0.9	−0.1 (−0.7, 0; −0.8, 0)	.**032**		3 months (T2)	7.1 ± 0.3	0.1 (−0.3, 0.3; −0.3, 0.4)	>.999	.948
	6 months (T3)	7.2 ± 0.9	−0.4 (−1.1, 0; −0.9, 0)	.**032**		6 months (T3)	7.0 ± 0.4	−0.1 (−0.6, 0.1; −0.6, 0.3)	.507	.821
CV %	Baseline (T0)	35.7 ± 6.6			CV %	Baseline (T0)	33.6 ± 5.4			.583
	1 month (T1)	34.2 ± 4.6	0.1 (−3.9, 1.9; −4.3, 1.8)	.433		1 month (T1)	32.5 ± 9.8	0.2 (−3, 3; −5.3, 2.8)	>.999	.624
	3 months (T2)	31.6 ± 4.3	−2.5 (−6.6, −1.4; −8.8, −0.6)	.**019**		3 months (T2)	33.6 ± 4.8	0.8 (−0.9, 2.1; −2.4, 3)	.502	.490
	6 months (T3)	31.5 ± 4.7	−3.6 (−5.9, −0.6; −9.2, 0.2)	.084		6 months (T3)	34.0 ± 4.7	0.7 (−1.6, 3.1; −1.9, 4)	.683	.226
HbA1c %; mmol/mol	Baseline (T0)	8.0 ± 1.3; 64.0 ± 14.0			HbA1c %; mmol/mol	Baseline (T0)	7.2 ± 0.9; 55.0 ± 10.0			.088
	1 month (T1)					1 month (T1)				
	3 months (T2)					3 months (T2)				
	6 months (T3)	7.4 ± 1.1; 57.0 ± 12.0	−0.3 (−0.9, 0.1; −1, −0.2)	.**021**		6 months (T3)	7.2 ± 0.7; 55.0 ± 8.0	−0.1 (−0.4, 0.1; −0.7, 0.3)	.360	.850
Sensor use %	Baseline (T0)	91.9 ± 8.9			Sensor use %	Baseline (T0)	87.1 ± 19.0			.907
	1 month (T1)	87.4 ± 13.7				1 month (T1)	90.3 ± 14.4			.506
	3 months (T2)	92.7 ± 8.8				3 months (T2)	93.0 ± 7.1			.817
	6 months (T3)	92.7 ± 10.0				6 months (T3)	90.1 ± 9.6			.361

Abbreviations: CHO, carbohydrates; CV, coefficient of variation; GMI, glucose management indicator; HbA1c, glycated hemoglobin; HL 95% CI, 95% confidence intervals for the Hodges-Lehmann estimate; IQR, interquartile range; SD, standard deviation; SG, sensor glucose; TAR, time above range; TBR, time below range; TIR, time in range. Statistically significant *P* values are highlighted in bold.

## Discussion

In this real-world observational study, switching from conventional multiple daily injections to the Smart MDI system was associated with progressive and sustained improvements in glycemic control over 6 months. The primary finding was a clinically meaningful increase in TIR and reduction in glycated hemoglobin values, accompanied by reductions in both hyperglycemia and hypoglycemia exposure. The magnitude of TIR improvement observed in our cohort (+9% at 6 months) is clinically relevant. International consensus recommendations have established TIR as a key CGM-derived metric associated with the risk of microvascular complications, with each 10% increase translating into meaningful reductions in complication risk and nearly 0.5% decrease in HbA1c levels (5, 19). In this context, the observed improvement corresponds to approximately 2 additional hours per day spent within the target glucose range. Notably, the increase in TIR was primarily driven by reductions in hyperglycemia, while hypoglycemia exposure decreased, aligning with consensus CGM targets that emphasize balanced glycemic improvements (5). Our findings are consistent with other recent real-world investigations on the Smart MDI system. In a 12-month retrospective study in adults with T1D, Longo et al reported sustained improvements in CGM-derived metrics following implementation of a connected insulin pen system, with significant reductions in hyperglycemia and stable hypoglycemia rates (20). Altogether, these data support the role of Smart MDI technology in optimizing glycemic profiles in routine clinical practice. The reduction in HbA1c was fair (−0.2%), but statistically significant, and consistent with the relatively good baseline glycemic control of the cohort. Prior studies have shown that CGM-derived metrics may detect clinically meaningful improvements not fully captured by HbA1c, particularly in real-world populations with near-target baseline values (21). These findings reinforce the complementary role of CGM metrics in evaluating technology-based interventions. When patients were stratified based on carbohydrate counting practice, no statistically significant between-group differences were observed. However, individuals not practicing carbohydrate counting experienced within-group improvements in TIR, TAR, mean SG, GMI, and HbA1c, whereas changes in the carbohydrate counting group were smaller and did not reach statistical significance, except for reduced TBR. This pattern may suggest that the structured feedback by “missed bolus” and “high glucose” alarms, and insulin dosing support provided by the Smart MDI system may partially compensate for the absence of formal carbohydrate counting strategies. Carbohydrate counting is an effective but cognitively demanding self-management approach requiring numeracy skills, accurate meal estimation, and consistent behavioral execution (22-25). Within this framework, the Smart MDI system may function as a structured decision-support tool that standardizes insulin administration and facilitates retrospective review of dosing patterns. Based on the within-group improvements observed among non-carbohydrate counting individuals, we hypothesize that such technologies may help mitigate disparities related to self-management skills. Importantly, improvements in hyperglycemia were not accompanied by increased hypoglycemia, suggesting that enhanced dosing structure did not compromise safety. This is particularly relevant when considering the implementation of technology in populations with less intensive educational support, where insulin over-titration and hypoglycemia risk could be concerns. Some limitations of this work should be acknowledged. The study was retrospective, single-center, and involved a relatively small sample size, limiting generalizability and statistical power, particularly for subgroup comparisons. We also acknowledge that the current sample size may reduce the statistical sensitivity to detect minor effects and thus observations may be preliminary. Although the study achieved sufficient post hoc power to detect the primary overall improvement in TIR owing to a moderate-to-large within-subject effect size, it was underpowered for subgroup comparisons. Moreover, the limited sample size restricted the possibility of applying more complex multivariable modeling approaches to adjust for potential confounding factors. Therefore, our findings, and especially subgroup observations, should be interpreted as exploratory and hypothesis-generating at this stage. Also, the transition from sensors with different accuracy, sampling frequency, alerts, and TIR calculation methods may have affected baseline CGM metrics, and thus it represents a limitation of the study. However, the improvement in TIR persisted after accounting for the potential impact of sensor transition, further strengthening the glycemic benefit observed during Smart MDI use. Finally, although the absence of a control group and the lack of imputation of missing observations may have introduced some selection bias, particularly at later time points, the within-subject longitudinal design allowed each participant to serve as their own reference, providing a real-world assessment of glycemic outcomes before and after Smart MDI initiation. Beyond the overall glycemic benefit, the differential pattern observed according to carbohydrate counting practice may suggest a potential role for Smart MDI technology as a structured support tool also in individuals not routinely applying formal carbohydrate counting strategies.

In conclusion, in adults treated with MDI therapy, transition to the Smart MDI system was associated with clinically meaningful glycemic improvements, with an increase in TIR and a reduction in hyperglycemia without increased hypoglycemia episodes. The observed response pattern in individuals not practicing carbohydrate counting warrants further investigation in larger prospective studies to clarify whether the Smart MDI system may provide targeted benefit in populations with less structured self-management approaches.

## Data Availability

The datasets used and/or analyzed during the current study are available from the corresponding author upon reasonable request.
